# Pulmonary infiltration as the initial manifestation of chronic lymphoproliferative disorder of natural killer cells: a case report and literature review

**DOI:** 10.1186/s12890-021-01457-y

**Published:** 2021-03-19

**Authors:** Jinjing Zhang, Pingping Wang, Xiaojing Yan

**Affiliations:** Department of Hematology, The First Affiliated Hospital of China Medical University, Shenyang, 110001 Liaoning China

**Keywords:** Chronic lymphoproliferative disorder of natural killer cells, Flow cytometry immunophenotyping, Bronchoalveolar lavage, Computed tomography

## Abstract

**Background:**

Chronic lymphoproliferative disorder of natural killer cells (CLPD-NK) is an extremely rare haematological disease. To the best of our knowledge, pulmonary infiltration in CLPD-NK has not been reported before. Our case study aimed to present the clinical characteristics, chest computed tomography (CT) findings, and flow cytometry immunophenotyping (FCI) results of an unusual case of migratory pulmonary infiltration in a patient with CLPD-NK.

**Case presentation:**

A 51-year-old female patient was admitted to our hospital on October 8, 2019. Eight months before this visit, she had been diagnosed with pneumonia in a community hospital with 1 month of low-grade fever and had recovered after oral antibiotic administration. During follow-up, the patient presented with persistent peripheral blood (PB) lymphocytosis and ground-glass opacities on lung CT scans without any symptoms and signs or any evidence of infectious, allergic or autoimmunity pulmonary diseases. Abnormal NK cells were identified in the PB, bone marrow and bronchoalveolar lavage fluid (BALF) using FCI in our hospital. Eventually, the patient was diagnosed with pulmonary infiltration of CLPD-NK. The patient had an indolent clinical course without symptoms, hepatosplenomegaly or palpable lymphadenopathy and did not receive any therapy. The patient has remained in a good performance status 13 months after the diagnosis.

**Conclusions:**

Our study described a unique case of pulmonary infiltration in a patient with CLPD-NK. The present case highlights the importance of FCI of the BALF in patients with lymphocytosis and pulmonary shadows to avoid misdiagnosis.

## Background

Within the rare group of large granular lymphocyte leukaemia (LGLL), chronic lymphoproliferative disorder of natural killer cells (CLPD-NK) is recognized as a provisional entity in the 2008 World Health Organization (WHO) classification of lymphoid neoplasms and retained this status in the 2016 revision [1]. It is characterized by persistent (> 6 months) clonal expansion of NK cells in the peripheral blood (PB) with an absolute NK cell count ≥ 2.0 × 10^9^/L [2]. The incidence of CLPD-NK is extremely low, and only approximately 300 cases have been studied, with the largest sample number being only 70 [3]. In most cases, patients with CLPD-NK are asymptomatic and present a chronic indolent disease course; a minority of patients present with fatigue and/or B symptoms, autoimmune disease, cytopenia, recurrent infections due to neutropenia, lymphadenopathy and hepatosplenomegaly [3]. However, to the best of our knowledge, pulmonary infiltration in CLPD-NK has never been reported. Here, we describe a case of pulmonary involvement resulting in migratory pulmonary shadows as the initial manifestation of CLPD-NK and review the relevant literature.

## Case presentation

**Fig. 1:**
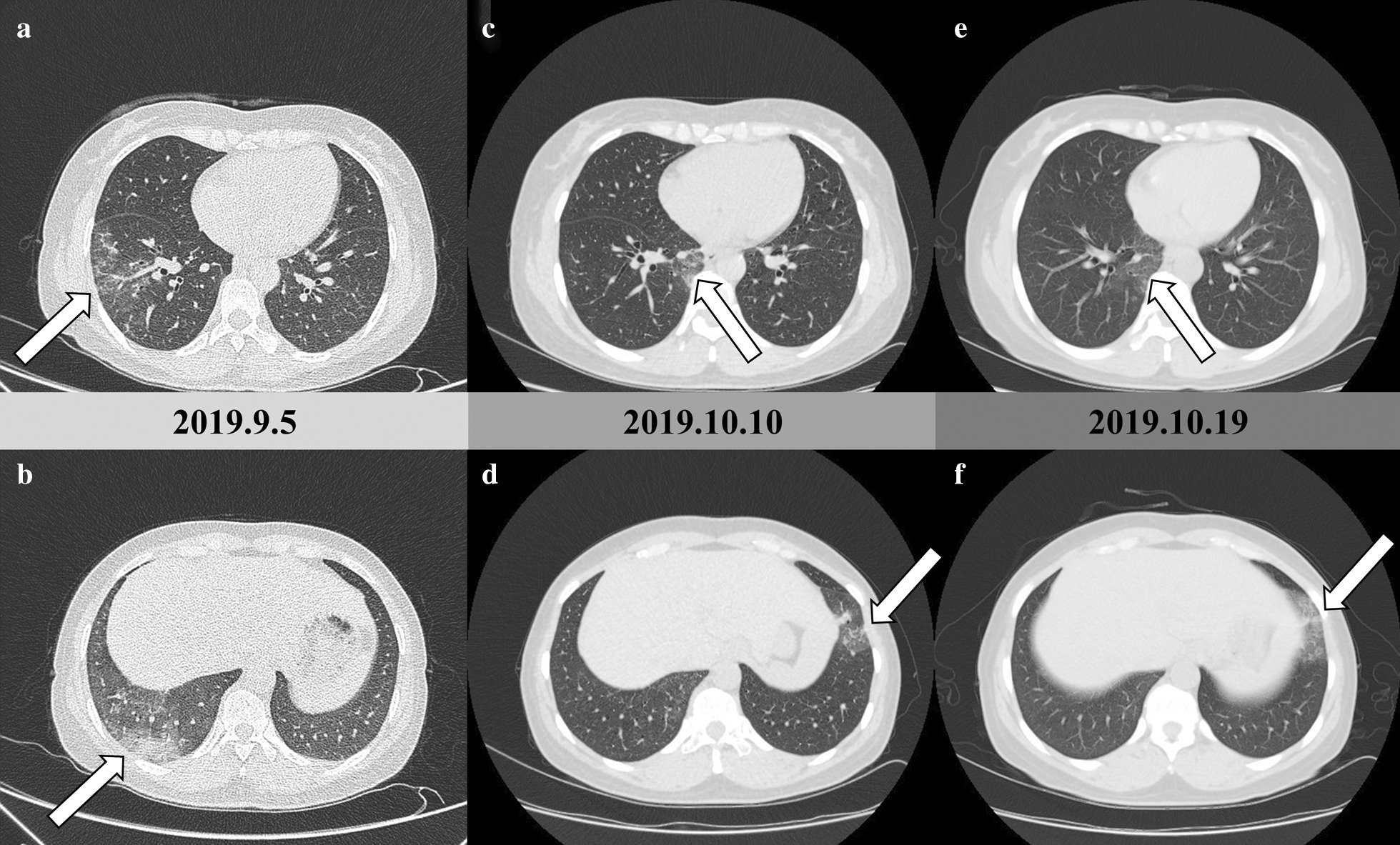
Representative chest CT images showing migratory pulmonary infiltration (white arrows) in a patient with CLPD-NK. **a**, **b** CT images captured on September 5, 2019, showing an inflammatory ground-glass opacity change in the lower lobe of the right lung. **c**, **d** CT images captured on October 10, 2019, showing the migratory pulmonary shadows with new ground-glass opacities in both of her lungs. **e**, **f** CT images captured on October 19, 2019, showing that the bilateral pulmonary ground-glass opacities not only persisted but also displayed an altered distribution

**Fig. 2:**
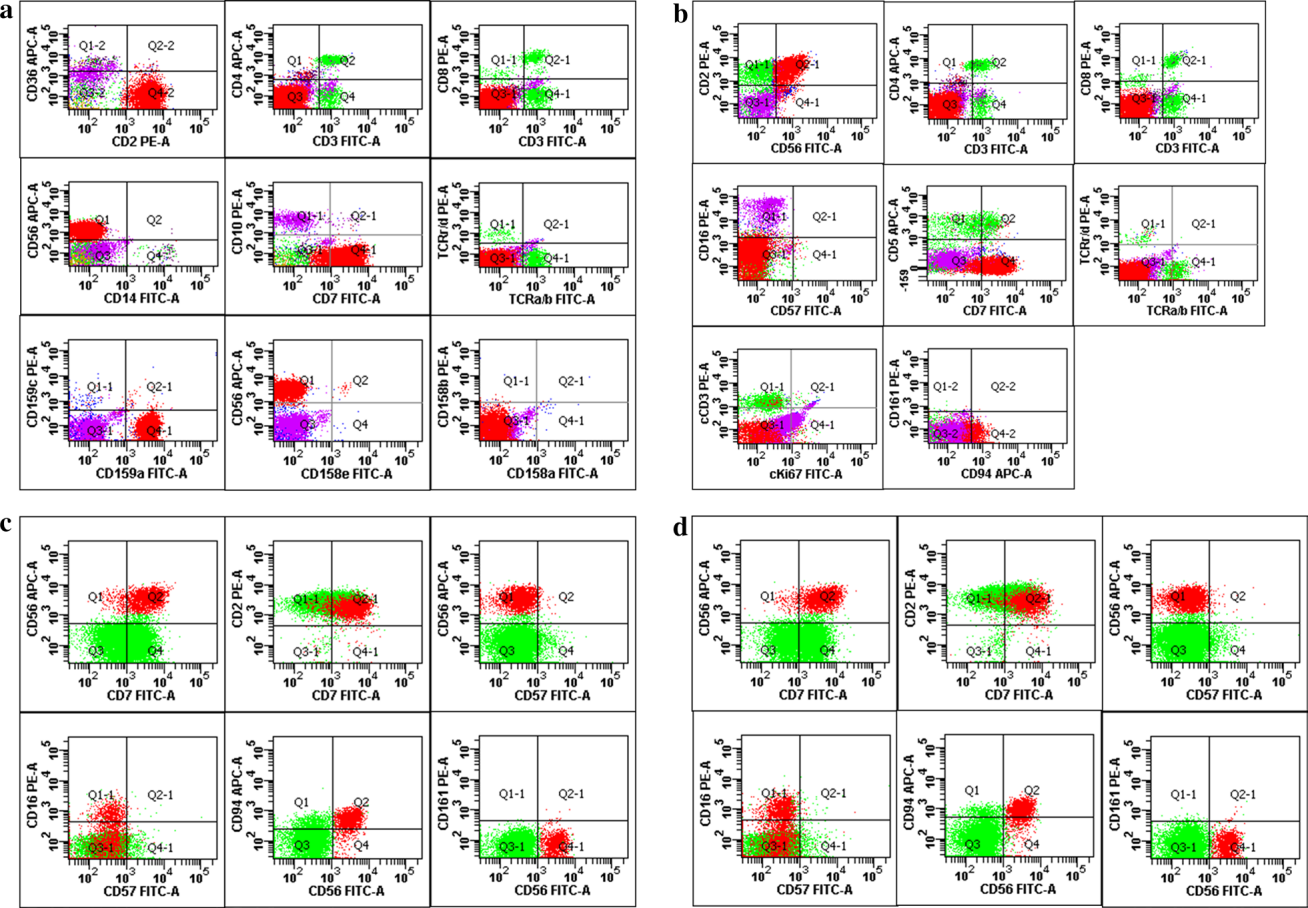
Representative scatter plots of FCI. A: PB, B: BM, C: BALF from the right lung, and D: BALF from the left lung. Abnormal NK cells are shown in red

A 51-year-old female patient was admitted to our hospital on October 8, 2019. She had been diagnosed with pneumonia in a community hospital with 1 month of low-grade fever and an increased lymphocyte count (5.2 × 10^9^/L) and ground-glass opacities on lung computed tomography (CT) scans in February 2019. The fever was relieved after oral antibiotic administration. Peripheral blood (PB) lymphocytosis was persistent and new ground-glass opacities on lung CT scans appeared at the 7-month follow-up (Fig. 1a, b). The patient was then referred to our hospital. A series of lung CT scans (October 10, 2019) suggested migratory pulmonary shadows with new ground-glass opacities in both of her lungs (Fig. 1c, d). The laboratory examination revealed an elevated white blood cell (WBC) count and lymphocyte count (WBC count, 17.43 × 10^9^/L; lymphocyte count, 12.97 × 10^9^/L). Renal and liver function were normal. The levels of inflammatory biomarkers, such as the erythrocyte sedimentation rate, C-reactive protein level, and procalcitonin level, were normal. The MycoDot test, an antiviral antibody series, an anti-*Mycoplasma pneumoniae* antibody test, a legionella pneumonia antibody test, a rheumatic antibody series (except an antinuclear antibody titre of 1:100+), an immunoglobulin test, and a cytomegalovirus DNA test were all negative. Slight elevations in β2-microglobulin (2.73 mg/L, reference, 0.7–1.8 mg/L) and lactate dehydrogenase concentrations (245 U/L, reference, 135–225 U/L) were observed. The patient was positive for Epstein-Barr virus (EBV-DNA) in the PB, with a level of 1.15 × 10^4^ copies/mL. Ultrasound indicated lymphadenopathy (the largest lymph node was 2.0 cm × 0.5 cm) with normal structures of the bilateral cervical, axillary and inguinal sites without hepatomegaly or splenomegaly. Due to the persistent lymphocytosis in the PB, flow cytometry immunophenotyping (FCI) of the PB was performed, and NK cells with an abnormal phenotype accounted for 66.6 % of the PB WBC population; this abnormal population mainly expressed CD56, CD7, CD2, and CD159a; partially expressed CD11c, CD38, and HLA-DR; and was negative for CD159c, CD158a, CD158b, CD158e, CD14, CD64, CD34, CD117, CD33, CD10, CD71, CD36, CD11b, CD3, CD8, CD4, cKappa, cLambda, CD94, CD161, CD57, CD1a, TCRαβ, TCRγδ, CD15, CD123, CD5, cKi67, cCD3, and CD16 (Fig. 2a). A PB smear showed large granular lymphocytes accounting for 90 % of the PB WBC cells. The aforementioned abnormal NK cells infiltrated the bone marrow (BM), as detected by a BM morphology examination and FCI, which showed 55.2 % and 59.0 % abnormal NK cells, respectively. The abnormal NK cells in the BM mainly expressed CD56, CD7, CD2, and CD45RO; partially expressed CD11b and CD94; and were negative for CD16, CD57, cKi67, cCD3, CD20, CD23, CD1a, CD3, TCRαβ, TCRγδ, Kappa, Lambda, CD19, CD5, CD45RA, CD161, CD4, and CD8 (Fig. 2b). Furthermore, a repeated examination of the chest CT results suggested that the bilateral pulmonary ground-glass opacities not only persisted but also exhibited an altered distribution (Fig. 1e,f). To clarify the nature of the lung lesions, bronchoalveolar lavage (BAL) was conducted, and FCI of the BAL fluid (BALF) revealed that abnormal NK cells accounted for 11.35 % and 12.41 % of the infiltrates in both lungs. The NK cells in the BALF mainly expressed CD56, CD7, CD2, and CD94; partially expressed CD16; and were negative for CD57 and CD161 (Fig. 2c, d). Results for T cell rearrangement and B cell rearrangement were all negative. Therefore, the patient was diagnosed with pulmonary infiltration in CLPD-NK. In our study, because this patient had an indolent clinical course without any symptoms or treatment indications, she did not receive any therapy and was monitored with a watch and wait approach. During the 13-month follow-up period, the patient has remained well without obvious symptoms, and the results of routine blood tests did not change significantly.

## Discussion and conclusions

LGLL, including T-cell LGLL (T-LGLL) (85 %), CLPD-NK (10 %) and aggressive NK-cell leukaemia (ANKL, 5 %), is a rare lymphoproliferative disorder characterized by the clonal expansion of large granular lymphocytes [1]. Chronic NK cell lymphocytosis was first identified in 10 patients with clinical features similar to those of patients with T-LGLL but less anaemia and neutropenia and more rheumatoid arthritis disorders in 1994 [4]. CLPD-NK, which is derived from the chronic expansion of CD3-NK cells in the PB, was proposed as a provisional entity in the 2008 WHO classification of lymphoid neoplasms, whereas T-LGLL is sustained by the proliferation of CD3 + T-cells. Although CLPD-NK and T-LGLL arise from different cell lineages, these two diseases share similar clinical manifestations with indolent and chronic courses, and ANKL is more aggressive than CLPD-NK and T-LGLL and has a poor prognosis [1]. In fact, most patients with CLPD-NK are symptomatic, and a number of associated disorders, such as autoimmune-associated diseases, have been described. However, to the best of our knowledge, pulmonary involvement in CLPD-NK has never been studied and could easily be misdiagnosed or ignored due to its extreme rarity and atypical imaging changes. In addition, we reviewed the CLPD-NK studies in the literature and reached conclusions regarding the clinical features of CLPD-NK.

Few studies on CLPD-NK have been published, and the largest case series is only 70, which included 37 cases from 3 previous studies [3]. In this report, we reviewed 322 patients reported in 13 main series on CLPD-NK with detailed clinical and laboratory features [2–14] (Table 1). A total of 49.2 % (124/252) of these patients had no symptoms at diagnosis, and the male/female sex ratio was 1.45 (176/121). Neutropenia was the most common laboratory abnormality, occurring in 41.9 % (116/277) of the patients. Anaemia and thrombocytopenia were observed in 26.2 % (77/294) and 17.0 % (47/277) of the patients, respectively. The following symptoms were recorded at the time of diagnosis: fatigue (24.6 %, 29/118), B symptoms (17.9 %, 26/145), splenomegaly (15.5 %, 46/297), infection (13.0 %, 18/138), lymphadenopathy (9.0 %, 18/199), and hepatomegaly (8.5 %, 17/199). A few of the patients with CLPD-NK had autoimmune disorders and other haematological diseases, such as unclassified arthritis, rheumatoid arthritis, polymyositis, polymyalgia, B-cell lymphoma, myelodysplastic syndrome, pure red cell aplasia, and skin diseases. In total, 37.1 % (13/35) of the patients with CLPD-NK were positive for EBV-DNA, and all of them were from Eastern countries; in contrast, EBV was described in only one study from a Western country, in which all the patients were negative [2]. A STAT3 gene mutation occurred in 20.9 % (31/148) of the patients. Among the 322 patients with CLPD-NK, none had lung involvement. In conclusion, CLPD-NK has a chronic clinical course with abnormal routine blood test results and atypical symptoms; therefore, immunophenotyping is important to provide a correct diagnosis.

**Table 1:** The clinical and laboratory characteristics of patients with CLPD-NK in previous studies [2–14]

Country/[reference]	Year	Patient number	Sex (M: F)	Age (median)	Neutropenia	Anaemia	Thrombocytopenia	Symptomatic	Fatigue	B symptoms	Hepatomegaly	Splenomegaly	Lymphadenopathy	Infection	EBV	STAT3 mutation
USA/[4]	1994	10	6: 4	60	3	2	2	7	NA	1	0	1	0	1	NA	NA
USA/ [5]	1999	16	14: 2	60.5 (7–78)	5	2	1	9	0	4	0	1	0	NA	NA	NA
Spain/ [6]	2004	26	11: 15	68 ± 17 (37–94)	11	12	7	3	NA	NA	1	4	3	1	NA	NA
Japan/ [7]	2005	19	10: 9	55 (14–69)	NA	NA	NA	NA	NA	NA	1	2	0	NA	6/7	NA
USA/ [8]	2012	50	28: 22	61 (27–90)	30	15	8	27	NA	NA	NA	6	NA	NA	NA	15/50
China/ [9]	2013	17	10: 7	65 (45–81)	NA	10	NA	NA	NA	3	1	1	1	NA	1/5	NA
China/ [10]	2014	8	2: 6	55.5 (28–75)	5	1	1	4	1	0	0	1	1	1	NA	NA
France/ [3]	2014	70	41: 29	61	23	13	14	36	18	10	9	10	4	10	NA	5/40
Italia/ [11]	2014	48	34: 14	61	13	4	3	23	NA	NA	NA	14	NA	NA	NA	4/48
China/ [12]	2016	9	5: 4	37 (16–62)	NA	NA	NA	NA	NA	NA	5	2	1	NA	5/9	NA
China/ [13]	2016	13	8: 5	54 (51–86)	7	7	2	9	4	4	0	0	4	0	1/13	NA
Italia/ [14]	2018	25	NA	62 (42–79)	10	2	3	NA	NA	NA	NA	NA	NA	NA	NA	NA
USA/ [2]	2018	11	7: 4	60 (25–89)	9	9	6	10	6	4	0	4	4	5	0/1	7/10
Total		322	176: 121		116/277 (41.9 %)	77/294 (26.2 %)	47/277 (17.0 %)	128/252 (50.8 %)	29/118 (24.6 %)	26/145 (17.9 %)	17/199 (8.5 %)	46/297 (15.5 %)	18/199 (9.0 %)	18/138 (13.0 %)	13/35 (37.1 %)	31/148 (20.9 %)

The diagnosis of CLPD-NK was based on an expansion of CD3-NK cells to an absolute count > 2.0 × 10^9^/L, which lasted for more than 6 months, and the exclusion of other clearly diagnosed disorders [1]. FCI plays an important role in diagnosing CLPD-NK because NK cells lack a clonal marker to distinguish between clonal and reactive progression. The typical FCI results for CLPD-NK are CD2+, CD3-, CD16+, and CD56+/-, and variable CD57 and killer cell immunoglobulin-like receptor (KIR) expression patterns have been described as surrogates of clonality in CLPD-NK. In addition, an FCI and clonality study of CLPD-NK indicated that increased expression of CD94 and HLA-DR might also be useful markers of NK cell clonality [14]. An increase in the NK cell count in the PB is observed in patients with some haematological diseases, including leukaemia, lymphoma, immune thrombocytopenic purpura, and myelodysplastic syndrome, and in patients with non-haematological diseases, such as solid tumours and skin diseases [15]. The NK cells in the PB of our patient were increased to a count greater than 2.0 × 10^9^/L for 8 months without evidence of other haematological or non-haematological diseases. In addition, negative expression of KIRs, including CD158a, CD158b, and CD158e, indicated the potential clonal expansion of the NK cells. Moreover, HLA-DR and CD94 were expressed on some NK cells in the PB and BM. Although the immunophenotyping results for our patient identified a CD3-CD16-CD56 + phenotype, which is more commonly observed in aggressive NK cells [12], the patient was asymptomatic and she was diagnosed with CLPD-NK. The patient remained well after a 1-year follow-up without any treatment.

The finding of haematolymphoid neoplasm infiltration in the BALF is quite rare, with only a few reports in the literature. Haematological malignancy with a pulmonary infiltrate was an infrequent finding, with an incidence of only 1 % (37/2977) of BAL specimens in a 22-year period, and another study of patients with acute leukaemia reported similar findings, with an incidence of leukaemic lung infiltration of 0.9 % (10/1130) [16]. Pulmonary involvement may occur in patients with haematological diseases such as leukaemia, lymphoma, plasmacytoma, and Langerhans cell histiocytosis. Primary pulmonary lymphoma (PPL) is extremely rare, accounting for only 0.4 % of all lymphomas and less than 0.5 % of all primary lung tumours. Secondary pulmonary lymphoma (SPL) is more frequent and defined as secondary involvement of pulmonary by systemic lymphomas. Mucosa-associated lymphoid tissue (MALT) lymphoma is the most common subtype of PPL, followed by diffuse large B-cell lymphoma (DLBCL), peripheral T-cell lymphoma, and follicular lymphoma (FL). Meanwhile, DLBCL is the most common subtype of SPL, followed by mantle cell lymphoma, MALT, FL, and Burkitt lymphoma [17]. As we presented, the symptoms and imaging manifestations of pulmonary infiltrates associated with haematological malignancies are nonspecific, mimicking an infection clinically and on CT findings. In fact, the imaging manifestations of pulmonary lymphoma vary, including consolidations, nodules, masses, diffuse intestinal diseases, cystic changes and pleural effusions [17, 18]. Therefore, BAL should be performed to determine the aetiology of pulmonary disease through analyses of morphological and microbiological parameters with few complications, even if patients are neutropenic [19]. However, the BALF from our patient was negative for microorganisms, such as *Mycobacterium tuberculosis*, fungi, and *Pneumocystis carinii*. Moreover, FCI of the BALF could be conducted to distinguish a malignancy from infectious or inflammatory progression with a relatively high accuracy [20]. FCI was able to identify malignant neoplastic cells in more than half of BALF specimens from patients with haematological malignancies, such as leukaemia, lymphoma and plasma cell myeloma, in a previous study [20]. Thus, FCI of the BALF is a useful diagnostic tool for the detection of haematological malignancy infiltration. In the present study, considering the migratory pulmonary imaging after antibiotic treatment, FCI of the BALF derived from the bilateral lungs was performed, showing immunophenotypic features consistent with those of the PB and BM. Therefore, the pulmonary migratory ground-glass density opacity was concluded to result from the lung infiltration of CLPD-NK, which has not been reported in the literature to the best of our knowledge.

In contrast to the poor prognosis of patients with acute leukaemia presenting with lung involvement [16], the patient with CLPD-NK we described with lung involvement still had an indolent disease, but we are not certain whether this disease will evolve into an aggressive form. Some reports have described the transformation of CLPD-NK with EBV infection into ANKL or NK cell lymphoma, and long-term follow-up is warranted in the future [7].

In summary, we described a unique pulmonary infiltration in a patient with CLPD-NK presenting with pulmonary ground-glass opacities as the initial manifestation. The present case study highlights that FCI of the BALF is a useful diagnostic tool for the detection of haematological malignancy infiltration. Importantly, we reviewed the CLPD-NK studies in the literature and reached conclusions regarding the clinical features of CLPD-NK based on the largest number of patients with CLPD-NK, which aims to improve physicians’ understanding of CLPD-NK.

## Data Availability

All data generated or analysed during this study are included in this published article.

## Abbreviations

ANKL: Aggressive NK cell leukaemia
BAL: Bronchoalveolar lavage
BALF: Bronchoalveolar lavage fluid
BM: Bone marrow
CLPD-NK: Chronic lymphoproliferative disorders of NK cells
CT: Computed tomography
DLBCL: Diffuse large B-cell lymphoma
EBV: Epstein–Barr virus
FCI: Flow cytometry immunophenotyping
FL: Follicular lymphoma
KIR: Killer cell immunoglobulin-like receptor
LGLL: Large granular lymphocyte leukaemia
MALT: Mucosa-associated lymphoid tissue
PB: Peripheral blood
PTCL: Peripheral T-cell lymphoma
PPL: Primary pulmonary lymphoma
SPL: Secondary pulmonary lymphoma
WBC: White blood cell
WHO: World Health Organization
